# Impact of Digital Literacy and Problematic Smartphone Use on Life Satisfaction: Comparing Pre- and Post-COVID-19 Pandemic

**DOI:** 10.3390/ejihpe12090091

**Published:** 2022-09-05

**Authors:** Busra Taskin, Chiho Ok

**Affiliations:** College of Business Administration, Kangwon National University, Chuncheon 24341, South Korea

**Keywords:** COVID-19 pandemic, digital literacy, problematic smartphone use, life satisfaction

## Abstract

Although the impact of digital literacy (DL) and problematic smartphone use (PSU) on life satisfaction was verified in previous literature, little is known about how the impact of two given variables can be differentiated during the COVID-19 pandemic situation. Thus, the purpose of this study was to empirically analyze whether the influence of DL and PSU on life satisfaction has changed as a consequence of the COVID-19 pandemic. We applied a cross-sectional study design to analyze data obtained from a nationwide survey on smartphone overdependence conducted in 2019 and 2020 by the South Korean Ministry of Science and ICT and the National Information Society Agency. Large-scale data obtained from 41,883 individuals were analyzed using hierarchical regression analysis. The results show that the positive relationship between digital literacy and life satisfaction was further strengthened post-COVID-19 rather than pre-COVID-19. In addition, the results suggest that the negative relationship between PSU and life satisfaction is further strengthened during post-COVID-19 rather than pre-COVID-19. The findings indicate that the roles of digital literacy and PSU are more important after the COVID-19 pandemic.

## 1. Introduction

Internet and smartphones are increasingly occupying more of people’s lives. Toward the end of 2021, the population of smartphone users reached four billion and is expected to increase with the rapid advancement in technology [1]. More people rely on smartphones to fulfill their daily responsibilities, and this trend is reported to be intensifying through the effects of COVID-19. According to OECD statistics, the internet usage rate in most countries increased compared to before COVID-19 [2]. Since the internet is a necessity for life due to the restriction of many offline activities during the COVID-19 era, many people use smartphones to work, study, share information, and develop social relationships more than they did prior to the pandemic [3,4,5,6,7]. However, the increase in the usage of smartphones has both positive and negative aspects that have been addressed in previous studies. Among them, we focused on the concept of digital literacy to explain the positive effect of smartphones and the concept of problematic smartphone use (PSU) to explain the negative effect of smartphones.

Digital literacy refers to the ability to search, evaluate, organize, and perform tasks through digital equipment and the internet in learning, work, and social life [8]. The expansion of information communication technology and the use of smartphones has required people to continuously train and acquire digital skills such as communicating, transacting, information handling, and problem-solving, which are essential in daily life. Therefore, the upsurge in digital literacy is attributed to the necessity of sustaining relationships and ensuring continuous communication among people [1]. Several studies argue that digital literacy has helped increase access to the information necessary to facilitate various tasks in organizations and at home. Kuss et al. [9] argue that digital literacy allowed the construction of new communication and interaction realities, resulting in a positive contribution to life situations. On the other hand, PSU refers to a decline in the ability to regulate smartphone use, and it is likely to cause excessive dependence symptoms such as lack of tolerance, withdrawal, escape, and craving [10]. Unlike digital literacy, which functions positively for the spread of an online society, numerous studies have highlighted that the PSU negatively impacts life satisfaction. These studies focus on the side effects of the excessive use of smartphones such as psychological illness, lack of sleep, and difficulties in thinking and concentration [11]. According to Van et al. [12], PSU is a form of psychological disorder characterized by behavioral dependence on smartphones, including other forms of digital media such as social media and the internet. The addictive nature of smartphones influences certain people to use them excessively without control and at the expense of other duties [13].

Although previous studies have demonstrated that digital literacy and PSU have opposing effects on life satisfaction, studies on the effects of the two variables comparing before and after COVID-19 remain non-existent. Consequently, it is necessary to further investigate how the effects of digital literacy and PSU have changed. In particular, given that the digital influence extended after COVID-19 has not diminished, a broader understanding of how the pandemic has changed the influence of online life-related factors, such as digital literacy and PSU, is required.

**Figure 1:**
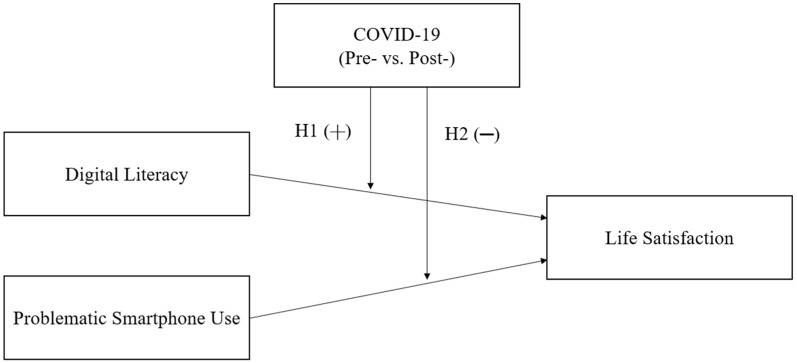
Research model.

Therefore, the purpose of this study is to empirically analyze whether the influence of digital literacy and PSU on life satisfaction has changed as a consequence of the COVID-19 pandemic. We integrated the data surveyed prior to COVID-19 (i.e., 2019) and during COVID-19 (i.e., 2020). Subsequently, we analyzed the moderating role of COVID-19 on the positive impact of digital literacy on life satisfaction and the negative impact of PSU on life satisfaction. This study provides implications for policymakers to identify whether the importance of digital literacy has been further emphasized or whether the seriousness of PSU has increased during COVID-19. Figure 1 shows the research model of this study. Specifically, we expect that the positive impact of digital literacy on life satisfaction will be further strengthened after COVID-19 rather than before COVID-19. Similarly, the negative impact of problematic smartphone use on life satisfaction will be expected to intensify after COVID-19 rather than before COVID-19.

## 2. Literature Review

### 2.1. Role of Digital Literacy on Life Satisfaction

Digital literacy positively impacts education with the remarkable rise of electronic learning [1,14]. With the advancement of technology, schools, colleges, and other institutions have introduced digital learning to allow students to attend classes through digital technologies. A significant percentage of students follow their lessons digitally through digital platforms while teachers and facilitators administer their classes and exams online. Research by Martínez-Alcalá et al. [15] suggests that online learning positively impacts information retention, and less time is taken to complete courses. This implies that the changes created by the pandemic might be long-lasting. By utilizing online learning platforms, teachers and students have unlimited access to video conferencing time, real-time co-editing of projects, auto-translation capabilities, and smart calendar scheduling that offer a reliable and effective learning experience. Currently, the adoption of e-learning is increasing in several regions globally, which implicates a positive impact on life situations.

Previous studies have shown that the higher the digital literacy, the more positive an impact smartphone usage will have on life satisfaction. Romero-Rodríguez et al. [16] note that a significant percentage of the global population resort to digital technology applications for leisure purposes. Currently, more people are using the internet for entertainment purposes and online shopping. As part of leisure time, individuals use their smartphones to video-call family and friends [15]. The use of the internet to make video calls helps boost interactions and ensure the overall well-being of family members and friends. Others resort to digital content sites such as Netflix, Showmax, and YouTube that offer relevant knowledge regarding lifestyles, documentaries, and fitness programs that have positive implications for the quality of life (QoL).

Martínez-Domínguez and Fierros-González [17] also highlight that teenagers and adolescents use their smartphones to play various video games that enhance their cognitive abilities, including reasoning, decision-making, and problem-solving. In addition to facilitating social interactions and enabling gaming activities, digital literacy has also played a significant role in online shopping. According to a study conducted by Van Velthoven and colleagues [12], over 45% of the global population reported having transacted business online during the COVID-19 period, with over half of the population making over three orders simultaneously.

Regarding the implications of smartphones on life satisfaction, previous studies generally show a positive relationship between the two. According to Herrero et al. [11], there has been an exponential increase in the number of people connecting online, making connections with friends, colleagues, and family, which has eventually helped strengthen interpersonal relationships. Social media platforms such as Facebook, Instagram, and WhatsApp help many people connect with friends and relatives globally [16].

### 2.2. Role of Problematic Smartphone Use on Life Satisfaction

Previous studies have indicated that increases in PSU correlate with increases in negative effects on life satisfaction. PSU negatively affects the QoL as the behavior is directly associated with high-stress levels among populations. Bilodeau et al. [14] have suggested that spending a significant amount of time on smartphones results in aggravated stress levels because users are faced with the fear of missing out. While investigating the correlation between PSU and stress, the study found that stress is directly associated with PSU severity, which accounts for the higher levels of stress experienced by people who overused their smartphones during the pandemic compared those who spent limited time on their phones. Fischer-Grote et al. [18], highlighted that stress negatively impacts life situations, as excessive use of smartphones agitates tension arising from lack of an appropriate means to attain social environment demands. Therefore, individuals are less likely to adapt and cope with new environmental demands than to experience despair [19]. Therefore, increasing stress levels in the population demonstrate that PSU negatively influences life situations.

Furthermore, comprehensive research indicates that problematic smartphone usage leads to poor relationships as it is increasingly challenging to build real relationships. According to Yang et al. [1], individuals who constantly engage their smartphones in isolation are exposed to the decreased time allocated to other social relations meant to build real relationships through in-person interactions. Through frequent use of smartphones, there is a tendency for online relationships to replace the in-person connections that are essential in developing high self-esteem, among other social interaction skills [13]. Another study by Kuss et al. [9], which links PSU with high levels of loneliness among users, concluded that online compulsions such as gaming, stock trading, and online shopping are currently rampant, limiting the necessity for face-to-face socialization and interactions. Furthermore, Park and Park [19] noted that PSU is often associated with an addiction that results in antisocial behaviors such as aggression that discourage the need to develop interpersonal relationships among populations.

Likewise, other studies report that PSU presented harmful effects on users’ physical health. According to Herrero et al. [11], individuals who spend the majority of their time online, playing games, or on social media platforms such as Facebook, Twitter, or YouTube are at risk of developing non-communicable diseases such as obesity, diabetes, and high blood pressure. Excessive use of smartphones reduces the time for physical activity. In most cases, smartphone users spend several hours enclosed in a particular setting which increases their rate of weight gain. Consequently, this increases the risks of developing non-communicable diseases [13]. While other factors such as the availability of fast foods also contribute to the development of diseases such as obesity and diabetes, a significant percentage of findings conclude that spending several hours on phones reduces the need to engage in physical activities such as sports [14].

Fatigue is also an indication of life dissatisfaction occasioned by the PSU disorder. Fatigue is associated with PSU, which affects how individuals work toward personal goals. Zhang et al. [20] investigated the connection between daytime fatigue, sleep quality, and PSU among medical students. Zhang et al. [20] utilized a web-based survey in Beijing involving 1016 participants between February and May 2020 and used multiple regression models and Spearman correlation coefficients to scrutinize the connection between PSU, daytime fatigue, and sleep deprivation. They found that 50% of the correspondents had PSU, and participants with PSU reported sleep disorder, mental weariness, and physical exhaustion. Moreover, the results showed that the indirect impact of PSU on mental tiredness and physical tiredness interceded through sleep quality accounted for 45% and 50% of the total result. Similarly, Mac Cárthaigh et al. [21] found a moderate relationship between sleep deprivation and PSU. Therefore, increased phone usage negatively affects biorhythm by increasing sleep disturbances and harming an individual’s well-being.

Furthermore, studies suggest high rates of anxiety among individuals struggling with PSU disorder. Anxiety has been linked to PSU and contributes to significant problems relating to social interactions at work and school. Yang et al. [22] systematically reviewed evidence from observational studies that associate PSU with anxiety, reduced sleep eminence, and hopelessness. The authors found an increased risk of anxiety, sleep deprivation, and despair in individuals experiencing PSU. Individuals experiencing psychopathological symptoms such as anxiety are likely to excessively utilize smartphones to combat anxiety. Consequently, this results in elevated nervousness and contributes to a vicious circle [23]. In particular, excessive internet use influences people to fear missing out as users anxiously check their smartphones. Stimulation from social media updates, calls, texts, and emails exposes an individual to chronic anxiety. Although these side effects are reversible, pressure can contribute to brain damage through non-pharmacological and pharmacological interventions [24]. The adverse side effects of anxiety include productivity loss due to reduced capability, the probability of industrial accident, and the risk of suicide. In this context, anxiety results in a feeling of life dissatisfaction, considering it affects individuals’ productivity in working toward their goals.

## 3. Hypotheses Development

### 3.1. The Moderating Role of COVID-19 in the Relationship between Digital Literacy and Life Satisfaction

Based on the above arguments, we predict that the effect of digital literacy on life satisfaction will be different depending on COVID-19. With the outbreak of the COVID-19 pandemic, the world is experiencing an increase in the percentage of people with access to smartphones and the internet. Several studies have demonstrated that the importance of digital literacy has been further emphasized since the pandemic [25,26,27,28,29]. These studies collectively agree that controlled exposure to smartphones contributes to digital literacy that positively impacts QoL. Since digital literacy will continue to increase access to the information necessary to facilitate a variety of tasks in organizations as well as at home even after the pandemic, it is easy to predict that it will contribute to psychological well-being. Therefore, we expect that the positive effect of digital literacy on life satisfaction has been further strengthened since the pandemic. Prior to the pandemic, there were positive influences from digital literacy; however, it is predicted that this influence will have been further amplified following the pandemic. Thus, we hypothesize the following.

**Hypothesis** **1.** *COVID-19 will moderate the relationship between digital literacy and life satisfaction such that the positive relationship between digital literacy and life satisfaction will be stronger post-COVID-19 rather than pre-COVID-19.*

### 3.2. The Moderating Role of COVID-19 in the Relationship between PSU and Life Satisfaction

Comparing the pre- and post-COVID-19 pandemic patterns, we predict that the effect of PSU on life satisfaction will be different. Cases of PSU were less reported prior to the COVID-19 pandemic, but they were prevalent during the pandemic involving uncontrolled and excessive usage, leading to anxiety, obsessive-compulsive behavior disorder, mental and physical fatigue, and sleep disturbance. In particular, recent studies related to PSU following COVID-19 have reported various evidence that COVID-19 amplifies severe PSU [23,30,31,32,33,34,35,36]. In addition, empirical results have shown that the PSU amplified by the pandemic negatively affects various areas of life, thereby hindering psychological well-being [34]. However, there is limited data comparing the impacts of PSU on life satisfaction or well-being and whether the changes are differentiated. Given the increased access to the internet globally and the high number of people owning smartphones after COVID-19, it is predicted that the negative impacts of PSU on human well-being will increase. Therefore, we expect the negative impact of PSU on life satisfaction to be more severe after COVID-19 than before COVID-19. Thus, we hypothesize the following.

**Hypothesis** **2.** *COVID-19 will moderate the relationship between problematic smartphone use (PSU) and life satisfaction such that the negative relationship between PSU and life satisfaction will be stronger post-COVID-19 rather than pre-COVID-19.*

## 4. Methodology

### 4.1. Participants

The data in this study were obtained from the 2019 and 2020 South Korean nationwide survey on smartphone overdependence conducted by the Ministry of Science and ICT and the National Information Society Agency [37]. This survey is conducted annually to secure data for policymaking on appropriate smartphone use. All items on the survey were translated into Korean by referring to the literature of each questionnaire. Smartphone users were defined as individuals who used the internet access feature of smartphones at least once a month. The 2019 survey targeted smartphone users aged 3–69 in all households nationwide as of September 2019. After stratified sampling based on the regional distribution of the population, the final sample of smartphone users consisted of 28,592 individuals in 10,000 households nationwide. Similarly, the survey was conducted in 2020, and 30,927 individuals in 10,000 households were surveyed.

In this study, the final sample was selected based on the following procedure. First, only individuals over the age of 20 were included. Although the survey included adolescents and children aged 3–19, these data were excluded from the analysis considering that confusion was caused related to lower life satisfaction in public education area at COVID-19. Second, the individuals whose data were missing were excluded from the analysis.

The final sample size consisted of 41,883 individuals. A total of 18,689 people were included in the 2019 survey, and 23,194 people were included in the 2020 survey. Of the 41,883 individuals, 20,380 were male (48.7%) and 21,503 were female (51.3%). The average age was 42.0 years, with a minimum of 20 years and a maximum of 69 years. The order of high utilization by content in 2019 (pre-COVID-19) was as follows: messenger (94.7%), watching movies, TV, and videos (89.9%), searching information (89.0%), news (88.2%), transportation service (84.5%), shopping (84.5%), music (80.2%), and games (67.4%). In 2020 (post-COVID-19), the orders were not significantly different, but the overall utilization rate had been increased such that messenger (95.2%), watching movies, TV, and videos (93.8%), news (91.4%), searching information (90.6%), transportation service (87.9%), shopping (87.7%), music (85.6%), and games (75.2%). Compared to before and after COVID-19, ‘watching movies, TV, and videos’ showed the largest increase in usage and this was followed by ‘shopping’ and ‘news.’

### 4.2. Instruments

Life satisfaction was measured using the Life Satisfaction Questionnaire. This contained one question about general life satisfaction and six related to domain-specific life satisfaction such as human relationships, work, health, consumption activities, leisure, and achievement. The responses to all questions were measured on the Likert 4-point scale (1 = very dissatisfied, 2 = dissatisfied, 3 = satisfied, and 4 = very satisfied). Cronbach alpha was 0.668.

Digital literacy was measured using a questionnaire consisting of six questions which is a short version of previously developed questionnaire [38]. Aspects of digital literacy used in this study include internet searching, content evaluation, communicating information, and creating digital content. The responses to all questions were measured on the Likert 4-point scale (1 = strongly disagree, 2 = disagree, 3 = agree, and 4 = strongly agree). Cronbach alpha was 0.796.

PSU was measured using the Smartphone Overdependence Scale (S-scale). The S-scale consists of three subscales, including self-control failure, salience, and serious consequences [19]. “Self-control failure” refers to a condition in which a person is unable to control their smartphone use according to self-set goals. “Salience” is the degree to which smartphone use becomes the most salient and important activity in one’s daily life. “Serious consequences” refers to negative physical, psychological, and social consequences resulting from PSU. Based on these concepts, 10 questions were asked to respondents in self-reported manner. The responses to all questions were measured on the Likert 4-point scale (1 = strongly disagree, 2 = disagree, 3 = agree, and 4 = strongly agree), with higher scores indicating more serious PSU. Cronbach alpha was 0.873.

COVID-19 was classified based on the data investigation date. Regarding South Korea, the first confirmed case of COVID-19 occurred in January 2020. Therefore, the 2019 data surveyed in August 2019 was considered pre-COVID-19. In this sense, the 2020 data surveyed in August 2020 was considered post-COVID-19. Consequently, pre-COVID-19 was coded as zero and post-COVID-19 as one, and this was used for analysis.

We controlled for several individual-level demographic variables that were found to affect life satisfaction, including gender, age, income, and education. In addition, gender was dummy-coded, where 1 = male, and 0 = female. Age is an individual’s age at the year of the survey. Income was measured based on the respondents’ average monthly income. It was measured at regular intervals, and coded from one (under one million KRW, approximately 756 EUR) to six (over five million KRW, approximately 3783 EUR). The education variable was controlled by calculating the number of years required for education based on each respondent’s final educational background.

### 4.3. Procedure and Data Analysis

As this study uses a continuous dependent variable (i.e., life satisfaction), the relationship between the variables was tested using regression analysis based on ordinary least square modeling. In addition, we conducted a hierarchical regression analysis that adds variables to the model to verify the moderating effect of COVID-19 in the relationship between digital literacy and life satisfaction, and between PSU and life satisfaction, respectively. Our reference model (Model 1) adds all the control variables including PSU and digital literacy. Model 2 includes an interaction term of digital literacy and COVID-19 to verify the moderating effect of COVID-19 in the relationship between two variables. Similarly, Model 3 includes an interaction term of PSU and COVID-19 to verify the moderating effect of COVID-19 in the relationship between two variables. Model 4 integrates the results of Model 2 and Model 3 by adding all variables including two interaction terms. In addition, we mean-centered all interaction terms to overcome multicollinearity. Furthermore, the bootstrapping procedures were carried out and the confidence intervals (95%) are reported for each step (from Model 1 to Model 4). All analyses were performed with the STATA 17.0 statistical program.

## 5. Analyses and Results

### 5.1. Descriptive and Correlation Analysis

Table 1 presents the means, standard deviations, and correlations of the variables. Life satisfaction positively correlates with digital literacy, gender, income, and education level, and negatively correlates with COVID-19 and age. Meanwhile, the age average of the final sample used in this study was 42.020 and the standard deviation was 11.699 (minimum value 20, maximum value 69). This contrasts with other studies dealing with the influence of smartphones on adolescents and younger generations. Moreover, since the skewness values do not exceed the absolute value of 3 and the kurtosis values do not exceed the absolute value of 8, all of variables can be regarded as a normal distribution [39].

### 5.2. Hierarchical Regression Analysis

We added the control variables and both of digital literacy and PSU in Table 2 into Model 1. Among the control variables, age (*b* = 0.001, *p* < 0.001), income (*b* = 0.029, *p* < 0.001), education level (*b* = 0.005, *p* < 0.001) are statistically significant and positively related to life satisfaction. However, the COVID-19 variable is statistically significant and negatively related to life satisfaction (*b* = −0.024, *p* < 0.001). These results demonstrate that for individuals, life satisfaction increases as age increases, income increases, and education level increases, however, overall satisfaction decreases due to COVID-19. Meanwhile, as predicted in previous studies, there is a positive relationship between digital literacy and life satisfaction (*b* = 0.310, *p* < 0.001). In addition, a negative relationship between PSU and life satisfaction was confirmed (*b* = −0.080, *p* < 0.001).

**Figure 2:**
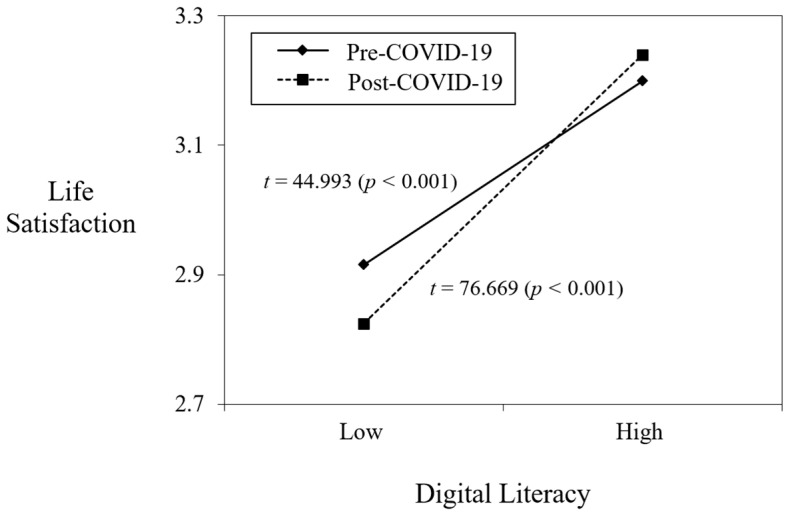
Moderating role of COVID-19 in the relationship between digital literacy and life satisfaction.

Hypothesis 1 posits that the COVID-19 variable moderates the positive relationship between digital literacy and life satisfaction. To test this, we added the interaction term of digital literacy and COVID-19 into Model 2 in Table 2. The results showed that the interaction term is positive and statistically significant (*b* = 0.112, *p* < 0.001). To check the pattern of interaction effect, we plotted the graph using statistics derived from the analysis. Figure 2 demonstrates that the positive relationship between digital literacy and life satisfaction was strengthened post-COVID-19 (slope difference test, *t* = 76.669, *p* < 0.001) rather than pre-COVID-19 (slope difference test, *t* = 44.993, *p* < 0.001). This implies that life satisfaction increased as an individual’s digital literacy increased during COVID-19 rather than before the pandemic. Therefore, Hypothesis 1 is supported.

**Figure 3:**
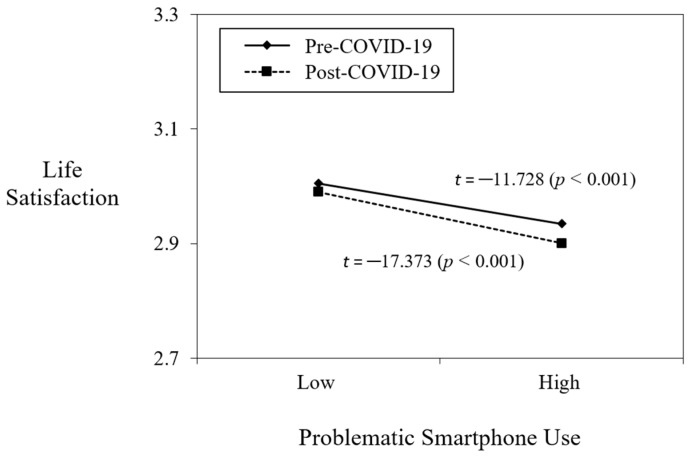
Moderating role of COVID_19 in the relationship between problematic smartphone use and life satisfaction.

Hypothesis 2 posits that the COVID-19 variable moderates the negative relationship between PSU and life satisfaction. To test this, we added the interaction term of PSU and COVID-19 into Model 3 in Table 2. The results show that the interaction term is negative and statistically significant (*b* = −0.019, *p* < 0.05). Similar to the first Hypothesis, to check the pattern of interaction effect, we plotted the graph using statistics derived from the analysis. Figure 3 demonstrates that the negative relationship between PSU and life satisfaction was strengthened post-COVID-19 (slope difference test, *t* = −17.373, *p* < 0.001) rather than pre-COVID-19 (slope difference test, *t* = −11.728, *p* < 0.001). This implies that life satisfaction decreased as an individual’s PSU increased during COVID-19 rather than before the pandemic. Therefore, Hypothesis 2 is supported.

## 6. Discussion

The results in this study aligned with previous research indicating that digital literacy can enhance individual well-being whereas PSU can harm individual well-being [14,15,16,18,19]. Since the proportion of life using smartphones has increased, the impact of online life on offline life has also been increasing [2]. Therefore, this study also confirmed that cultivating digital literacy capabilities and preventing and reducing PSU behavior are important for individual well-being.

The finding of this study is also consistent with previous studies that show digital literacy and PSU are more important in the face of COVID-19. Several empirical studies have demonstrated that the importance of digital literacy has been further emphasized since the pandemic [25,26,27,28,29]. Recent studies related to PSU following COVID-19 have also reported various evidence that COVID-19 amplifies severe PSU [23,30,31,32,33,34,35,36]. This study supports the empirical evidence that the importance of digital literacy and PSU after COVID-19 has increased by showing that both variables have a greater impact (positively, and negatively, respectively) on individual well-being.

In particular, this study is in line with previous studies in that the results have been proven in South Korea, where the proportion of smartphone use is very high [5,19,24]. As of 2022, South Korea’s smartphone penetration rate is 97%, the highest in the world [37]. In other words, if the proportion of smartphones in other countries increases more and more as in Korea, it can be predicted that the influence of digital literacy and PSU will also increase more and more.

### 6.1. Theoretical and Practical Implications

This study contributes to previous studies in that the digital literacy increases the effect on life satisfaction under certain environmental conditions (i.e., during COVID-19). However, COVID-19 is not the only thing that shows the importance of digital literacy. After COVID-19, schools introduced online classes to avoid face-to-face encounters, companies dramatically increased online shopping, and the communities and societies exchanged a lot of information through the Internet, not face-to-face. Under such circumstances, there will be various third context factors that influence the role of digital literacy. Therefore, future research should investigate how the influence of digital literacy on various areas of an individual’s life is combined with various contextual factors such as individual characteristics, social structure, and national culture.

In addition, this study contributes to previous studies as it verified the negative effects of PSU by comparing them before and after the pandemic. This study highlighted the severity of PSU caused by COVID-19 and indicated that its negative impact on life satisfaction has increased. Furthermore, given that the negative effect of PSU has increased since COVID-19, the importance of PSU management has also increased. Therefore, attempts to identify various determinants affecting PSU following the COVID-19 pandemic should be extensively undertaken.

In terms of practical implications, this study suggests that digital literacy should be strengthened, and the importance of policies and education to prevent PSU should be reiterated. In particular, this study highlights the fact that the need for the systematic management of the two variables is increasing. Schools and local communities need to more actively undertake education or campaigns that can increase people’s digital literacy. Simultaneously, it is necessary to introduce policies and programs to improve the early detection and treatment of PSU in order to prevent the personal and social problems it causes. Although this study did not identify which social group is more problematic regarding PSU following COVID-19, future studies should identify this in order to target the group and seek practical alternatives for them.

### 6.2. Limitation and Future Research

This study has several limitations. First, it is difficult to generalize the study results because each country may have different smartphone penetration, utilization, and COVID-19 situation. Therefore, in future studies, it is necessary to re-verify the similarity of these results in contexts other than South Korea. Second, there is a limitation of common method bias because the questionnaire was surveyed based on single-sourced and self-reported methods. In order to more clearly estimate the causal relationship, investigation with a time interval between the independent variable and the dependent variable is required. Third, it is necessary to reconfirm the research results with panel data that has been repeatedly investigated for the same sample. Although this study integrated and compared the data at two points in time (2019 and 2020), there may be differences in direct comparison because they are not the same sample. Therefore, it is necessary to analyze whether the pattern of digital literacy or PSU has changed before and after COVID-19, or whether the effect on life satisfaction is differentiated in future studies. Finally, the respondents’ smartphone usage patterns were not reflected. The pattern may vary depending on what kinds of contents, such as games, social networking sites, or shopping, they spend their time using. However, this study was unable to consider the difference in smartphone usage time or application. Consequently, future studies should investigate how the pattern of digital literacy or PSU varies depending on the usage pattern.

## 7. Conclusions

We found that the impact of digital literacy and PSU on life satisfaction was greater after the COVID-19 pandemic situation. Therefore, theorists should find out what effective determinants can increase digital literacy and reduce PSUs in future studies. Furthermore, from a practical point of view, it is useful to focus more on the both variables in order to improve individuals’ life satisfaction. Further education is required to improve digital literacy in schools and communities. In addition, campaigns for the appropriate level of digital literacy in the public sector should also be undertaken. Activities to treat and enlighten adolescents and adults with symptoms of PSU should be continuously undertaken. In particular, countries where the penetration rate of smartphones is high but the importance of digital literacy and PSU is overlooked should realize their importance and promote related activities. These activities will increase individual life satisfaction and contribute to reducing side effects, which will aid the development of our online (as well as offline) society.

## Figures and Tables

**Table 1:** Descriptive statistics and correlation matrix.

Variables	1	2	3	4	5	6	7	8
1. LS	(0.668)							
2. DL	0.394 ***	(0.796)						
3. PSU	−0.001	0.235 ***	(0.873)					
4. COVID-19	−0.025 ***	0.001	−0.004	1.000				
5. Gender	0.046 ***	0.021 ***	0.091 ***	−0.001	1.000			
6. Age	−0.120 ***	−0.201 ***	−0.398 ***	0.117 ***	0.044 ***	1.000		
7. Income	0.096 ***	0.004	0.076 ***	0.035 ***	0.005	−0.016 **	1.000	
8. Education level	0.160 ***	0.134 ***	0.374 ***	−0.080 ***	0.105 ***	−0.509 ***	0.128 ***	1.000
Mean	3.007	2.695	1.872	0.553	0.486	42.02	2.988	14.434
S.D.	0.431	0.582	0.506	0.497	0.499	11.699	0.904	2.15
Skewness	−0.320	−0.355	0.552	−0.216	0.053	0.080	0.677	−0.838
Kurtosis	2.829	2.899	2.903	1.047	1.003	2.220	3.779	2.718

Note 1. LS = life satisfaction; DL = digital literacy; PSU = problematic smartphone use. Note 2. The number in parentheses means the Cronbach alpha value. *N* = 41,883. * *p* < 0.05, ** *p* < 0.01, *** *p* < 0.001.

**Table 2:** The results of hierarchical regression analyses predicting LS.

Variables	Model 1		Model 2		Model 3		Model 4	
	b	s.e.	b	s.e.	b	s.e.	b	s.e.
Constant	2.091 ***	0.023	2.276 ***	0.025	2.071 ***	0.024	2.238 ***	0.026
	[2.046; 2.138]		[2.226; 2.327]		[2.023; 2.120]		[2.187; 2.290]	
Gender (1 = male, 0 = otherwise)	0.004	0.003	0.005	0.003	0.004	0.003	0.005	0.003
	[−0.003; 0.012]		[−0.002; 0.013]		[−0.003; 0.012]		[−0.002; 0.013]	
Age	0.001 ***	0.0002	0.001 ***	0.0002	0.001 ***	0.0002	0.001 ***	0.0002
	[0.001; 0.002]		[0.001; 0.002]		[0.001; 0.002]		[0.001; 0.002]	
Income	0.029 ***	0.002	0.029 ***	0.002	0.029 ***	0.002	0.029 ***	0.002
	[0.025; 0.033]		[0.026; 0.034]		[0.026; 0.034]		[0.026; 0.034]	
Education Level	0.005 ***	0.001	0.005 ***	0.001	0.005 ***	0.001	0.005 ***	0.001
	[0.003; 0.007]		[0.003; 0.007]		[0.003; 0.008]		[0.003; 0.007]	
COVID-19	−0.024 ***	0.003	−0.025 ***	0.003	−0.024 ***	0.003	−0.025 ***	0.003
	[−0.032; −0.017]		[−0.033; −0.018]		[−0.032; −0.017]		[−0.033; −0.018]	
DL	0.310 ***	0.003	0.244 ***	0.005	0.309 ***	0.003	0.237 ***	0.005
	[0.302; 0.317]		[0.233; 0.255]		[0.303; 0.317]		[0.226; 0.248]	
PSU	−0.080 ***	0.003	−0.079 ***	0.003	−0.069 ***	0.005	−0.049 ***	0.005
	[−0.088; −0.072]		[−0.087; −0.072]		[−0.081; −0.058]		[−0.061; −0.037]	
COVID-19 * DL			0.112 ***	0.006			0.123 ***	0.006
			[0.099; 0.126]				[0.110; 0.137]	
COVID-19 * PSU					−0.019 *	0.007	−0.052 ***	0.007
					[−0.034; −0.004]		[−0.068; −0.037]	
F-value	1236.69 ***		1124.86 ***		1083.02 ***		1005.86 ***	
R-squared	0.1713		0.1769		0.1714		0.1778	
Adj. R-squared	0.1712		0.1767		0.1713		0.1776	

Note 1. LS = life satisfaction; DL = digital literacy; PSU = problematic smartphone use. Note 2. The numbers in square brackets were 95% confidence interval. *N* = 41,883. * *p* < 0.05, ** *p* < 0.01, *** *p* < 0.001.

## Data Availability

The data of this study is available to anyone under the permission of the National Information Society Agency in South Korea (https://www.nia.or.kr; accessed on 21 July 2022).
